# Electrophysiological signatures of anxiety in Parkinson’s disease

**DOI:** 10.1038/s41398-024-02745-x

**Published:** 2024-01-27

**Authors:** Sahar Yassine, Sourour Almarouk, Ute Gschwandtner, Manon Auffret, Peter Fuhr, Marc Verin, Mahmoud Hassan

**Affiliations:** 1 MRC Brain Dynamic Unit, Nuffield Department of Clinical Neurosciences, University of Oxford, Oxford, United Kingdom; 2 University of Rennes, LTSI - U1099, F-35000 Rennes, France; 3 Behavior & Basal Ganglia, CIC1414, CIC-IT, CHU Rennes, Rennes, France; 4 Neuroscience Research Centre, Lebanese University, Faculty of Medicine, Beirut, Lebanon; 5 Dept. of Neurology, Hospitals of the University of Basel, Basel, Switzerland; 6 Institut des Neurosciences Cliniques de Rennes (INCR), Rennes, France; 7 France Développement Electronique, Monswiller, France; 8 Movement Disorders Unit, Neurology Department, Pontchaillou University Hospital, Rennes, France; 9 School of Science and Engineering, Reykjavik University, Reykjavik, Iceland; 10 MINDIG, F-35000 Rennes, France

**Keywords:** Neuroscience, Psychiatric disorders

## Abstract

Anxiety is a common non-motor symptom in Parkinson’s disease (PD) occurring in up to 31% of the patients and affecting their quality of life. Despite the high prevalence, anxiety symptoms in PD are often underdiagnosed and, therefore, undertreated. To date, functional and structural neuroimaging studies have contributed to our understanding of the motor and cognitive symptomatology of PD. Yet, the underlying pathophysiology of anxiety symptoms in PD remains largely unknown and studies on their neural correlates are missing. Here, we used resting-state electroencephalography (RS-EEG) of 68 non-demented PD patients with or without clinically-defined anxiety and 25 healthy controls (HC) to assess spectral and functional connectivity fingerprints characterizing the PD-related anxiety. When comparing the brain activity of the PD anxious group (PD-A, N = 18) to both PD non-anxious (PD-NA, N = 50) and HC groups (N = 25) at baseline, our results showed increased fronto-parietal delta power and decreased frontal beta power depicting the PD-A group. Results also revealed hyper-connectivity networks predominating in delta, theta and gamma bands against prominent hypo-connectivity networks in alpha and beta bands as network signatures of anxiety in PD where the frontal, temporal, limbic and insular lobes exhibited the majority of significant connections. Moreover, the revealed EEG-based electrophysiological signatures were strongly associated with the clinical scores of anxiety and followed their progression trend over the course of the disease. We believe that the identification of the electrophysiological correlates of anxiety in PD using EEG is conducive toward more accurate prognosis and can ultimately support personalized psychiatric follow-up and the development of new therapeutic strategies.

## Introduction

Anxiety is a highly prevalent psychiatric comorbidity in Parkinson’s Disease (PD), affecting up to 31% of the patients [1], which is three times more prevalent than the general elderly population [2]. It can emerge at any stage of the disease, and be present even during the prodromal stage [3, 4]. The clinical presentation of this disorder can include various subtypes [1, 5, 6] such as General Anxiety Disorder, non-episodic and episodic anxiety, panic attacks, and social phobia, which can worsen motor symptoms [7–9] and cognitive functioning [10–13] and decrease the quality of life of patients [14, 15]. Moreover, anxiety in PD comorbid often with other psychiatric symptoms such as depression and apathy [16, 17], and the extensive overlap in their relevant features has hindered their clinical dissociation [18]. As a result, anxiety in PD is often underdiagnosed [1, 19] and undertreated [20] yet limited scientific attention has been given to understand its underlying pathophysiology.

Non-invasive neuroimaging techniques are increasingly used to investigate the neural mechanisms of anxiety in PD [17, 21]. Positron emission tomography (PET) and anatomical magnetic resonance imaging (MRI) studies have associated the anxiety in PD with reduced metabolism and cortical thickness in several subcortical regions including the amygdala, as well as in the bilateral anterior cingulate and prefrontal cortex [21–25]. Using fMRI resting state studies, functional disruptions in emotional-related cortical and subcortical regions were reported to correlate with anxiety symptoms [21, 26–28].

Electroencephalography (EEG) has been growingly employed to uncover the neural correlates of complex neuropathologies [29, 30], such as neuropsychiatric disorders [31, 32]. Providing direct measures of the neural activity, EEG has proven to be a valuable, non-invasive and cost-effective tool for biomarkers development. To date, only one study has compared anxious and non-anxious PD patients using EEG, revealing frequency-related spectral and functional disruptions, mainly in the frontal cortex, that characterize the anxiety in PD [33]. Yet, the use of EEG in case-control longitudinal studies to assess the neural correlates of anxiety in PD is still missing.

Here, we used High-Density (HD)-EEG recordings to excerpt the electrophysiological signatures of anxiety in PD by comparing the spectral patterns and functional networks of anxious PD patients (PD-A) to non-anxious PD patients (PD-NA) and healthy controls (HC). We quantified the spectral and network signatures in terms of electrophysiological scores and assessed their relationship with clinical scores of anxiety over the course of the disease.

## Materials and methods

### Participants

The study population, described in our previous studies [30, 34], was composed of PD patients and healthy controls (HC) enrolled from the Movement Disorders Clinic of University Hospital of Basel (city of Basel, Switzerland) as a part of a longitudinal study approved by the local ethics committees (Ethikkommission beider Basel, Basel; Switzerland; EK 74/09). The diagnosis of PD was based on the United Kingdom Brain Bank criteria for idiopathic Parkinson’s disease [35]. To be included in the study, patients had to meet specific criteria including a Mini-Mental State Examination (MMSE) score of 24 or above, no previous history of vascular or demyelinating brain disease, and sufficient proficiency in the German language. All participants provided written informed consent and were fully informed of the nature of the study. Included patients underwent neurological, neuropsychological, neuropsychiatric and EEG examinations at baseline (BL) and follow-up after a mean interval of 3 years (3Y) and 5 years (5Y).

As we focused on anxiety in PD, only participants that presented anxiety assessments were included in this study. Accordingly, 68 non-demented PD patients (22 females, age: 66.4 ± 8.3) and 25 HC (10 females, age: 66.4 ± 4) were selected at BL. As for the 3Y follow-up, the sample size was set to 42 PD patients (14 females, age: 70.5 ± 7.9) and 17 HC (9 females, age: 68.9 ± 6). At 5Y, 34 PD patients (13 females, age: 71.1 ± 6.8) and three healthy controls (1 female, age: 65.7 ± 4.1) presented anxiety assessments and were included in the main study cohort. Table S1 of the supplementary materials represent the main demographic, clinical and neuropsychological characteristics of the analysis cohort.

### Neurological, neuropsychological, and neuropsychiatric evaluations

Basic neurological and comprehensive neuropsychological examinations were carried out in all the participants. Patients were evaluated on their regular dopaminergic medication (“ON” state) and the use of antidepressant and anxiolytics treatments was reported. The global cognitive score was assessed using the Montreal cognitive assessment score [36] (MoCA), and patients were classified as with or without mild cognitive impairment (MCI) according to the Movement Society Task Force Level II criterias described in Litvan et al. [37]. Depression was measured using the Beck Depressive Inventory, second edition [38] (BDI-II, German version) and apathy was assessed based on the Apathy Evaluation Scale [39] (AES, German version).

Anxiety symptoms were evaluated using the German version of the Beck Anxiety Inventory [40] (BAI), a 21 items self-rating scale. Each item is evaluated on a four-point Likert scale ranging from 0 to 3 (e.g., not at all; a little; moderate; or many). The total score ranges from 0 to 63 with higher scores representing increased symptoms severity. Leentjens et al. [41] have validated the use of BAI in PD. As a score higher than 13 has been identified to show clinically significant anxiety, this cut-off was considered to divide the PD patients into two groups: PD patients with clinically relevant anxiety PD-A (N = 18) and PD patients without anxiety PD-NA (*N* = 50).

### EEG acquisition and preprocessing

Resting state EEG data were recorded for all participants using a HD-EEG system with 256 channels (Netstation 300, EGI, Inc., Eugene, OR). Participants were asked to relax, close their eyes and stay awake while seated in a comfortable chair for 12 minutes. The sampling rate was set to 1000 Hz. The raw EEG data were segmented into epochs of 40 seconds each and the first epoch of each recording was discarded from the analysis. As described in our previous study [34], epochs were preprocessed automatically using the open-source toolbox Automagic [42]. Briefly, signals are subjected to band-pass filtering between 1 and 45 Hz, followed by the electrooculography (EOG) regression on 17 frontal electrodes to eliminate ocular artefacts. This step reduces the final number of channels to 239, which are mapped to four lobes of interest: frontal, parietal, temporal and occipital (see Table S2 and Fig. S1 of the supplementary materials). Subsequently, bad channels exhibiting high variance (higher than 20 μV) or amplitude exceeding ± 80 μV are identified and interpolated. Finally, the artefact-free epochs were sorted according to their quality metrics and only the best six were retained for the rest of the analysis.

### Power spectral analysis

The Welch method [43] was used to estimate the power spectrum of signals at the scalp level. It consisted of computing a modified periodogram using the Hamming window with one second duration and 50% overlap to obtain the absolute power spectral density (PSD). The relative power spectrum was then computed by normalizing each value of the absolute power spectrum by the total sum of the powers at each frequency of the EEG broadband (1-45 Hz). A [239 × 45] relative power features at the scalp level were thus obtained and used for further analysis.

### Functional connectivity analysis

The functional brain networks were estimated using the source-connectivity method [44]. First, the inverse problem was solved to reconstruct the dynamics of the cortical brain sources: the EEG channels and the MRI template (ICBM152) were co-registered, a realistic head-model was built using the OpenMEEG [45] toolbox, and the weighted Minimum Norm Estimate (wMNE) method [46] was applied on the cortical signals. The obtained source signals were then averaged into the 210 regions of interest (ROIs) of the brainnetome atlas [47], which are mapped into seven cortical lobes of interest: Prefrontal (PFC), Motor (Mot), Parietal (Par), Temporal (Tmp), Occipital (Occ), Limbic (Lmb) and insular (Ins). Their affiliation is presented in Table S3 of the supplementary materials. Afterwards, the phase synchrony between different ROIs was computed using the Phase Locking Value (PLV) method [48] and the dynamic functional connectivity matrices were estimated for six different EEG frequency bands: delta (1–4 Hz), theta (4–8 Hz), alpha1 (8–10 Hz), alpha2 (10–13 Hz), beta (13–30 Hz), and gamma (30–45 Hz). Those matrices were ultimately averaged across time and trials and their 21,945 unique connections [= 210 × 209/2] in each frequency band were used for further analysis.

As for the longitudinal analysis, functional connectivity networks were similarly estimated at 3Y and 5Y but without including gamma frequency band due to abnormal noise within this band in most of the patients at 5Y.

### Statistical analysis

The statistical differences in demographic and clinical characteristics between the PD-A, PD-NA and HC groups were examined using the one-way analysis of variance (ANOVA). The chi-square test (for the categorical variables) and the independent samples t-test (for the continuous variables) were applied to examine the difference between the PD-A and PD-NA groups. Covariates such as age, sex, education levels and variables that showed significant differences between groups were included in the subsequent analysis.

Our main objective was to compare EEG-based features of the PD-A group to both PD-NA and HC groups. To accomplish this three-group comparison, we employed a two-step statistical process. First, we used a permutation-based non-parametric analysis of covariance (Perm-ANCOVA) to examine statistical differences in the relative power spectrum [239 channels x 45 frequencies] and functional connectivity networks [21945 connections x 6 bands] of the three groups at BL. We used 1000 permutations to identify the first set of significant power/connectivity features (*p* < *0.05*). As we were interested in identifying the features that predominantly represent the PD-A group, we defined two conditions: the PD-A_high_ condition, where the power/connectivity values of the PD-A group were significantly higher than both the PD-NA and HC groups (PD-A > PD-NA & PD-A > HC), and the PD-A_low_ condition, where the power/connectivity values of the PD-A groups were significantly lower than both other groups (PD-A < PD-NA & PD-NA < HC). Next, the second step of the process involved applying a two-tailed between-groups Wilcoxon test (corrected for multiple comparisons, *p* < *0.0167*) on the previous set of statistically significant features. Significant features that meet one of the above conditions were subsequently retained and considered as electrophysiological signatures of anxiety in PD.

### Anxiety signature scores and correlation analysis

In order to quantify the electrophysiological signature of anxiety in PD, two separate signature scores were defined: the spectral signature score (SSS) and the network signature score (NSS). The SSS is delineated as the ratio between the power indexes (PI) of the two previously defined conditions: PD-A _high_ → *PI*_*high*_ and PD-A _low_ → *PI*_*low*_:

1 $${SSS}=\frac{{{PI}}_{{high}}}{{{PI}}_{{low}}}$$

where *PI* is the mean relative power of the significant channels in the significant frequency slices and defined as:

2 $${PI}=\frac{1}{{Nslices}}\,\mathop{\sum }\limits_{i=1}^{{chan}}\mathop{\sum }\limits_{j=1}^{{freq}}{SigPower}(i,j)* {PS}{D}_{{rel}}(i,j)$$

Where *SigPower* is a [239 × 45] binary matrix obtained from the statistical analysis representing the significant channels and their corresponding frequency slices, *PSD*_*rel*_ is the [239 × 45] matrix of the relative power features, *chan* is the total number of channels, *freq* is the total number of examined frequencies and *Nslices* is the total number of significant slices in *SigPower*.

Similarly, the NSS is defined as the ratio between the network indexes (NI) obtained from the significant edges of both conditions: PD-A _high_ → *NI*_*high*_ and PD-A _low_ → *NI*_*low*_:

3 $${NSS}=\frac{{{NI}}_{{high}}}{{{NI}}_{{low}}}$$

where *NI* is the mean connectivity of the significant edges (connections) in all frequency bands:

4 $${NI}=\frac{1}{{Nconnections}}\,\mathop{\sum }\limits_{i=1}^{{con}}\mathop{\sum }\limits_{j=1}^{{band}}{SigNetwork}(i,j)* W(i,j)$$

Where *SigNetwork* is a [21945 × 6] binary matrix obtained from the statistical analysis representing the significant connectivity features in each frequency band, *W* is the [21945 × 6] matrix containing the functional connectivity features, *con* is the total number of unique connections, *band* is the total number of EEG frequency bands and *Nconnections* is the total number of significant connections in *SigNetwork*.

Pearson’s correlation was used to examine the relationship between the electrophysiological signature scores (SSS/ NSS) and the clinical anxiety score (BAI) not only at BL but also at 3Y and 5Y to assess their prediction capacity.

## Results

### Participant’s characteristics

Table 1 shows the demographic and clinical characteristics of the participants. No significant differences were found neither in the demographic features (age, sex and education) between all groups nor in the clinical assessments and the antiparkinsonian medication doses between the PD groups. Evidently, the BAI score was significantly discriminable between the three groups (*p* < 0.0001). Also, both depression score (BDI-II) and apathy score (AES) presented a significant difference between groups (*p* < 0.001) and significantly correlated with the BAI score. Therefore, they were both considered as covariates in the statistical analysis.

**Table 1:** Longitudinal demographic and clinical characteristics of the three groups expressed as: mean (standard deviation).

	Baseline			3 years			5 years	
	PD-A (*N* = 18)	PD-NA (*N* = 50)	HC (*N* = 25)	PD-A (*N* = 15)	PD-NA (*N* = 27)	HC (*N* = 17)	PD-A (*N* = 8)	PD-NA (*N* = 26)
Demographic								
Age (y)	65.7 (8)	66.6 (8.4)	66.6 (4)	71.3 (9.9)	70.1 (6.6)	70.2 (4.1)	70.6 (9)	71.2 (6.2)
Sex (M/F)	12/6	34/16	15/10	10/5	18/9	11/6	5/3	16/10
Education (y)	14.9 (3.7)	14.7 (3.1)	14.2 (2.9)	16.3 (2.8)	14 (3.1)	14.1 (3.3)	15.6 (1.9)	14.1 (3.3)
Clinical								
Disease duration (y)	4.9 (5.6)	5.3 (5.1)	–	7.6 (3.9)	7.5 (5.1)	–	9 (2.1)	10.8 (5.7)
MoCA (/30)	26 (2.8)	26 (2.3)	26.6 (2.7)	23.3 (5)	26.3 (2)	26.8 (2.5)	25.3 (6.4)	25.9 (2.9)
MCI (Y/N)	5/13	17/33	–	7/8	8/19	–	1/7	9/15
Medication								
LEDD (mg/day)	616 (461)	664 (470)	–	653 (402)	676 (463)	–	758 (232)	529 (341)
Antidepressant (Y/N)	5/13	8/42	–	4/11	5/22	–	2/6	2/24
Anxiolytics (Y/N)	4/14	8/42	–	1/14	4/23	–	0/8	1/25
Neuropsychiatric tests								
BAI (/63)	20.3 (7.2)^a,b^	6.2 (3.9)^a,b^	2.4 (3.2)^a^	19.8 (4.8)^a,b^	6.9 (3.5)^a,b^	2.5 (2.9)^a^	19.5 (4.9)^b^	6.5 (3.5)^b^
BDI-II (/63)	11.2 (5.1)^a,b^	6.4 (4.1)^a,b^	2.6 (2.5)^a^	11.5 (4.2)^a,b^	5.6 (3.8)^a,b^	1.8 (1.7)^a^	14.6 (7.7)^b^	4 (2.6)^b^
AES (/63)	17.5 (10)^a^	6 (7)^a^	1(4)^a^	36.8 (8.2)^b^	28.8 (4.8)^b^	–	36.6 (8.3)^b^	29.3 (6.6)^b^

*PD-A* PD patients with anxiety, *PD-NA* PD patients without anxiety, *HC* healthy controls, *y* years, *M/F* Male/Female, *MoCA* Montreal Cognitive Assessment, *MCI (Y/N)* Mild Cognitive Impairment (yes/no), *LEDD* Levodopa Equivalent Daily Dose, *BAI* Beck Anxiety Inventory score, *BDI-II* Beck Depression Inventory, second edition score, *AES* Apathy Evaluation Scale.

^a^Indicate significant *p*-value of ANOVA between the three groups (*p* < 0.001).

^b^Indicate significant *p*-value of t-test between PD-A and PD-NA groups (*p* < 0.05).

### Spectral signature of anxiety in PD

**Fig. 1:**
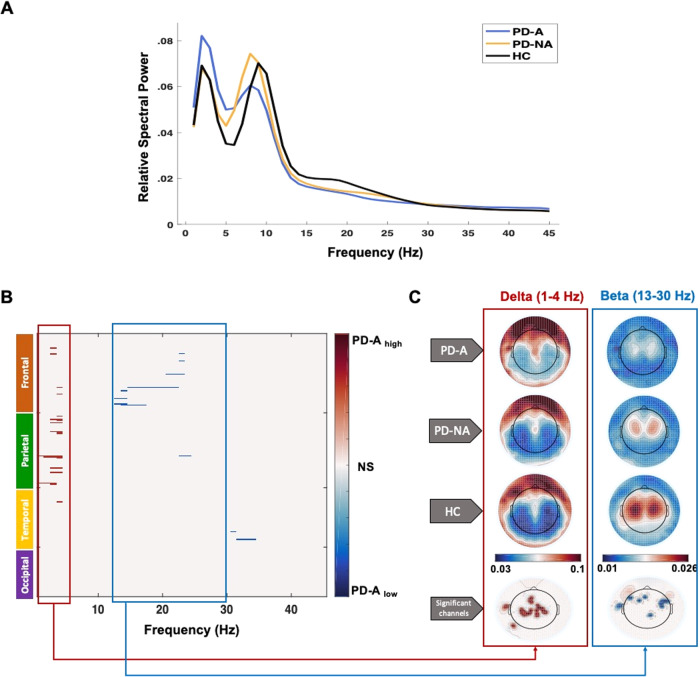
Spectral signature of anxiety in PD. **A** the relative power spectra of the three groups: PD patients with anxiety (PD-A) and without anxiety (PD-NA) and healthy controls (HC). **B** Significant channels and frequency slices of the PD-A_high_ (PD-A > PD-NA, HC) condition in red and PD-A_low_ (PD-A < PD-NA, HC) condition in blue. **C** Cortical topography of the relative spectral power of the relevant frequency bands (delta in PD-A_high_ and beta in PD-A_low_) for the three groups and the corresponding spatial distribution of the significant channels (significant channels are marked in red for delta band and in blue for beta band). NS no-significance.

The average relative spectral power over all EEG channels for the three groups is illustrated in Fig. 1A. Our statistical analysis on the overall [239 × 45] spectral features at BL allowed us to identify the spectral signature of anxiety in PD. This includes the EEG channels with their corresponding frequency slices where the PD-A group has either significantly higher or significantly lower spectral power than both the PD-NA and HC groups (PD-A_high_ and PD-A_low_ conditions). For the PD-A_high_ condition, results showed 20 significant channels with corresponding frequency slices mainly within the delta band (between 1 and 4 Hz). Those channels were presented notably in the parietal and frontal lobes. As for the PD-A_low_ condition, 11 channels mainly located within the frontal lobe and presenting significant frequency slices between 13 and 25 Hz (within the beta band) were revealed (Fig. 1B). The cortical topography of the relative spectral power observed in each group for the relevant frequency slices (delta and beta bands) of both conditions, along with the spatial distribution of the corresponding significant channels are illustrated in Fig. 1C.

### Network signature of anxiety in PD

Owing to uncovering the network signature of anxiety in PD, we repeated the same statistical analysis described above on the 21945 unique functional connectivity features of the six examined frequency bands. This resulted in identifying for each frequency band, a significant network of both hyper-connectivity edges (PD-A_high_ condition: where the connectivity in PD-A is significantly higher than in PD-NA and HC) and hypo-connectivity edges (PD-A_low_ condition: where the connectivity in PD-A is significantly lower than in PD-NA and HC).

**Fig. 2:**
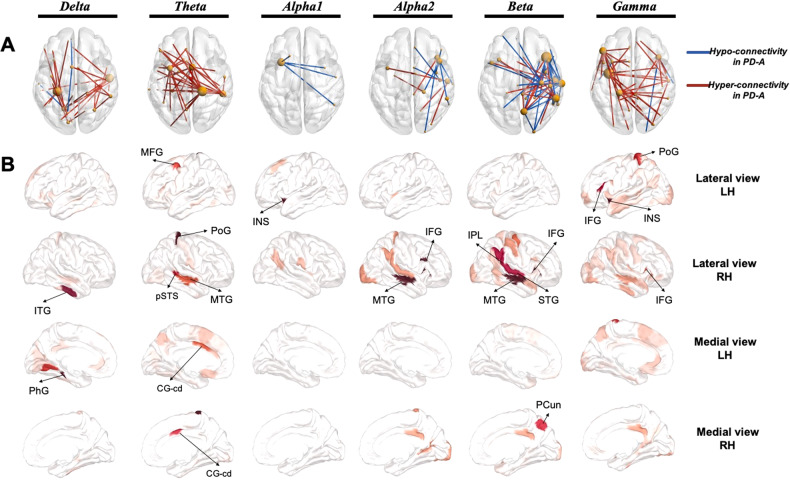
Network signature of anxiety in patients with PD. **A** Significant networks of the different investigated frequency bands. The networks were thresholded for visualization purposes. Edges presenting hyper-connectivity in PD-A are illustrated in red (PD-A_high_) and those presenting hypo-connectivity in PD-A are illustrated in blue (PD-A_low_). **B** Highest degree regions (thresholded for visualization purposes) represented with different views (lateral and medial) of the left hemisphere (LH) and right hemisphere (RH). ITG Inferior Temporal Gyrus, PhG Parahippocampal Gyrus, MFG Middle Frontal Gyrus, PoG Postcentral Gyrus, pSTS Posterior Superior Temporal Sulcus, MTG Medial Temporal Gyrus, CG-cd Cingulate Gyrus caudodorsal region, INS Insula, IFG Inferior Frontal Gyrus, IPL Inferior Parietal Lobule, STG Superior Temporal Gyrus, PCun Precuneus.

Results showed that hyper-connectivity networks characterizing the PD-A group were dominant in delta, theta and gamma bands, while hypo-connectivity networks were more prevalent in alpha and beta bands (Fig. 2A). Further investigation of brain regions with the greatest number of connections (highest degree regions) in these significant networks revealed that regions within the temporal lobes were present in almost all bands. In particular, the middle temporal gyrus (MTG) appeared in theta, alpha2 and beta bands. Additionally, the inferior frontal gyrus (IFG) was featured in networks of higher frequencies (alpha2, beta and gamma). Regions within the salience network (SAN) were among the most prevalent in theta (the caudodorsal region of the anterior cingulate gyrus (CG-cd)), and in alpha1 and gamma (the insula (INS)) (Fig. 2B).

**Fig. 3:**
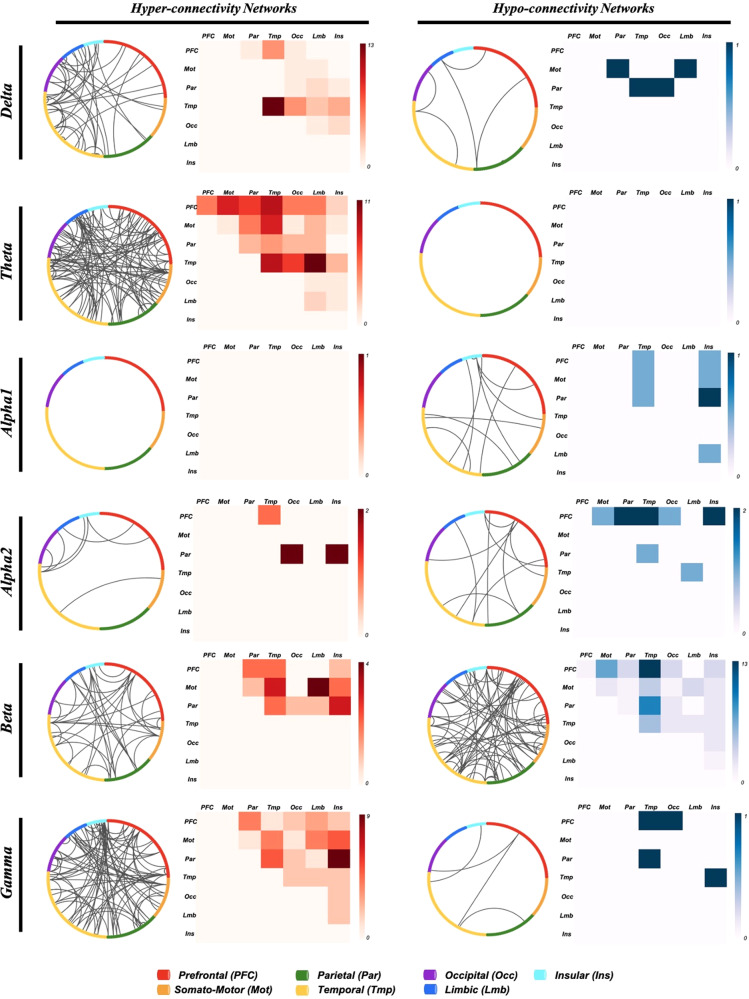
Representation of the network signature of anxiety in patients with PD. Circular plots (left) and matrix plot (right) of the significant networks in Delta, Theta, Alpha1, Alpha2, Beta and Gamma frequency bands. Red and blue shades represent the number of connections in the hyper-connectivity networks and hypo-connectivity networks respectively.

Upon examining the interactions between the cortical lobes within these networks, we observed that the hyper-connectivity networks displayed dense functional connections primarily between the temporal, limbic and insular lobes. Specifically, the most prominent connections were temporo-temporal in delta, temporo-limbic in theta, motor-limbic in beta and insular-parietal in gamma bands. Regarding the hypo-connectivity networks, the insular lobe exhibited denser connections in the alpha band, with insular-parietal connections being the most dominant in alpha1 and insular-frontal connections prevailing in alpha2. Additionally, fronto-temporal hypo-connections were prevalent in beta bands. To illustrate these findings, circular and matrix plots displaying the interaction between the lobes of interest in the hypo/hyper connectivity networks across all bands are illustrated in Fig. 3.

### Electrophysiological signature scores of anxiety

**Fig. 4:**
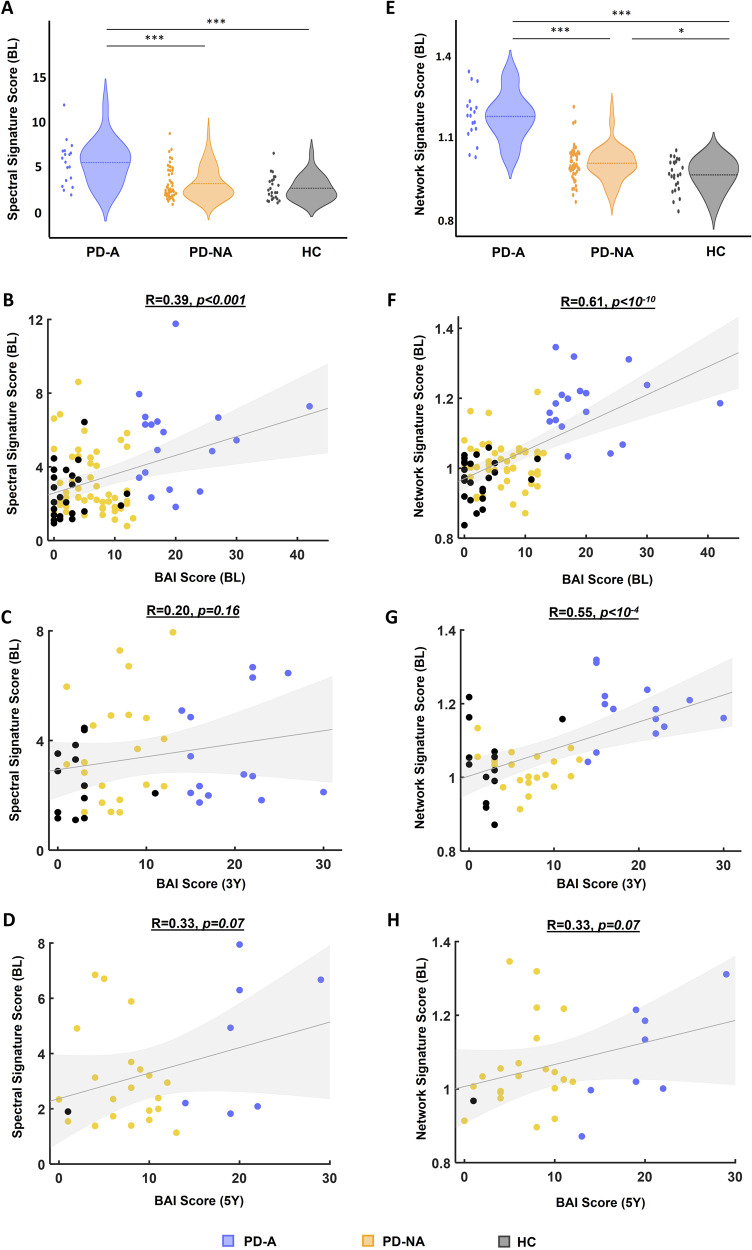
Spectral Signature Score (SSS) and Network signature score (NSS) of anxiety and their relationship with the BAI score. Distribution of the (**A**) SSS and (**E**) NSS between the three groups: PD patients with anxiety (PD-A), without anxiety (PD-NA) and healthy controls (HC). Relationship between the SSS/NSS at BL and BAI score: (**B**, **F**) at BL, (**C**, **G**) at 3Y, (**D**, **H**) at 5Y. ***p < 0.001, **p < 0.01, *p < 0.05 (*p*-values are corrected using Bonferroni for multiple comparisons).

In order to appraise the spectral signature of anxiety in PD and associate it with clinical scores, we computed the SSS as the ratio between the average power of the significant channels/slices of the PD-A_high_ condition over the PD-A_low_ condition. Consequently, this resulted in investigating the spectral ratio between delta and beta bands. Results showed that the SSS of the PD-A group was significantly higher than both the PD-NA and HC groups (*p* < 0.001, Bonferroni corrected, Fig. 4A). This SSS was significantly correlated with the BAI score (*R* = 0.39, *p* < 0.001) of the participants at BL (Fig. 4B). The SSS computed at BL remained positively correlated with the BAI scores at 3Y (*R* = 0.20, *p* = 0.16, Fig. 4C) and at 5Y (*R* = 0.33, *p* = 0.07, Fig. 4D), but without being statistically significant.

Similarly, we also investigated the association between the network signature score (NSS) and the clinical evaluation of anxiety. This score represents the ratio between the average connectivity of the hyper-connectivity edges and that of the hypo-connectivity edges in all frequency bands. Results showed that the NSS was significantly higher in the PD-A group compared to both PD-NA and HC groups (*p* < 0.001, Bonferroni corrected, Fig. 4E). Further, the NSS computed from BL networks showed a strong correlation with the BAI score measures at BL (*R* = 0.61, *p* < 10^−10^, Fig. 4F) and at 3Y (*R* = 0.55, *p* < 10^−4^, Fig. 4G). A positive trend toward significance was also shown at 5Y (*R* = 0.33, *p* = 0.07, Fig. 4H), demonstrating notable predictive ability.

### Longitudinal evolution of anxiety networks, anxiety clinical scores, and anxiety electrophysiological scores

To investigate the progression of anxiety-related EEG patterns and their capacity to reflect clinical scores, we chose to track the longitudinal changes in average connectivity within the distinct hypo/hyper-connectivity networks for the PD-A and PD-NA groups separately, along with corresponding changes in the BAI score and NSS score. This choice was made because the NSS computed at 3Y showed a significant difference between PD-A and PD-NA, unlike the SSS, which was not able to differentiate between the three groups (Fig. S2 of the supplementary materials). Among the 18 PD-A and 50 PD-NA patients at BL, we specifically selected patients who had longitudinal clinical assessments at 3Y (12 PD-A and 24 PD-NA) and at 5Y (8 PD-A and 13 PD-NA).

**Fig. 5:**
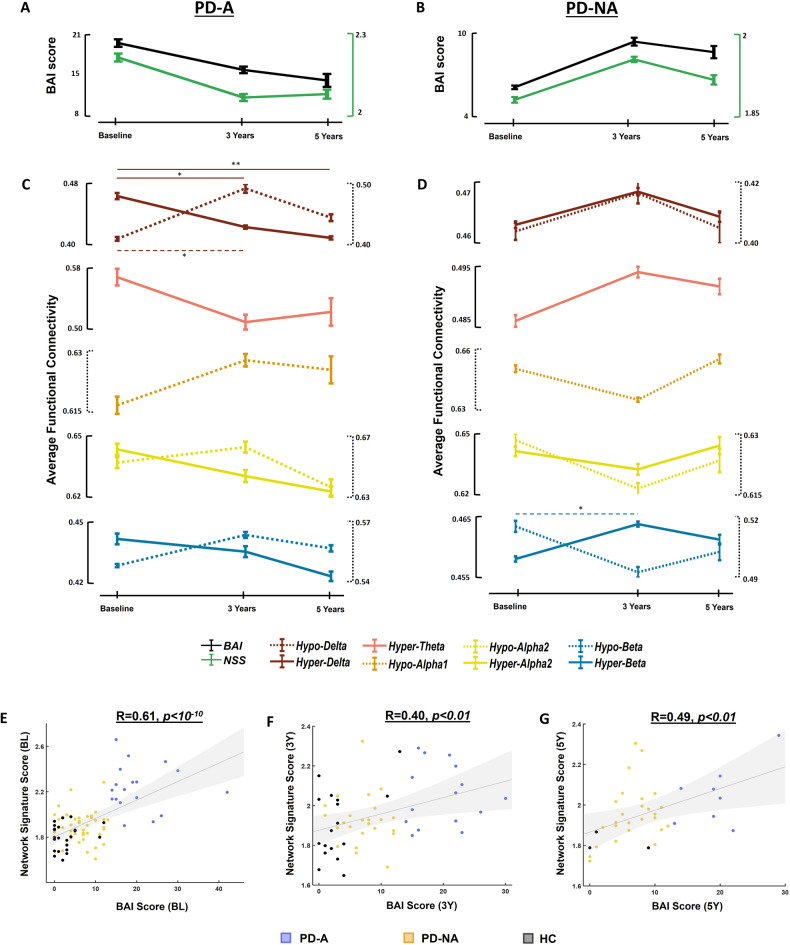
Longitudinal progression of anxiety networks, anxiety clinical scores and anxiety electrophysiological scores. Longitudinal progression of the clinical scores of anxiety (BAI score) and the Network Signature Score (NSS) between BL, 3Y and 5Y for (**A**) PD patients with anxiety (PD-A) and (**B**) PD patients without anxiety (PD-NA). Longitudinal progression of the average functional connectivity of significant hyper/hypo-connectivity networks of (**C**) PD-A and (**D**) PD-NA. Relationship between the NSS of all participants and their clinical BAI scores (**E**) at BL, (**F**) at 3Y, and (**G**) at 5Y. **p < 0.01, *p < 0.05.

Our results showed that the average BAI score of the PD-A group patients decreased over time between BL, 3Y, and 5Y. This same decreasing trend was observed in the longitudinal NSS, mainly between BL and 3Y (Fig. 5A). Regarding the band-specific network patterns of anxiety, the same decreasing trend between BL and 5Y was observed in the average functional connectivity of the hyper-connectivity networks of delta, theta, alpha2, and beta, along with an opposite increasing trend corresponding to the hypo-connectivity networks of delta, alpha1, and beta, mainly between baseline and 3Y (Fig. 5C). As for the patients of the PD-NA group, their BAI scores showed an increase over time, mainly between BL and 3Y. This increase was also observed in the NSS as depicted in Fig. 5B. Furthermore, the average functional connectivity of the hyper-connectivity networks in delta, theta, and beta followed this same increasing trend, whereas an opposite decreasing trend was also observed for the hypo-connectivity networks of alpha1, alpha2, and beta (Fig. 5D).

We also computed the NSS derived from the functional connectivity networks of all participants at BL, 3Y and 5Y (excluding gamma networks for comparisons purposes), and investigated their relationship with the clinical scores of anxiety. Consistent with the significant correlation observed at BL (*R* = 0.61, *p* < 10^−10^, Fig. 5E), the NSS remained significantly correlated with the BAI score at 3Y (*R* = 0.40, *p* < 0.01, Fig. 5F), and at 5Y (*R* = 0.49, *p* < 0.01, Fig. 5G) suggesting relevant prediction capacity.

## Discussion

In the present study, we aimed to identify the electrophysiological signatures of PD-related anxiety using resting state HD-EEG. While controlling the presence of other neuropsychiatric symptoms (depression and apathy), we showed that anxiety in PD is characterized by increased delta power -at the scalp level- in the frontal and parietal lobes as well as reduced beta power in the frontal lobe. Our functional connectivity analysis revealed that hyper-connectivity networks dominate in delta, theta and gamma bands while hypo-connectivity networks are more present in alpha and beta bands, with the frontal, temporal, limbic and insular lobes exhibiting the majority of significant connections. Electrophysiological scores computed from the network signatures distinguished the PD-A group from both PD-NA and HC groups longitudinally. These EEG-based scores followed the progression of the clinical scores of anxiety and correlated with them at BL, as well as at 3Y and 5Y, demonstrating predictive capacity.

Our spectral analysis at the channel-frequency level allowed for an accurate spatial-spectral mapping of the power features that characterize the PD-A group compared to both the PD-NA and HC groups. The increased power in delta and decreased power in low beta (13-20 Hz) are consistent with the global spectral patterns observed in the single previous EEG study that compared PD-A and PD-NA patients [33]. Our findings were also consistent with spectral patterns observed in anxious non-parkinsonian subjects. Increased delta power in frontal and parietal lobes was reported to characterize induced anxiety in obsessive compulsive-disorder patients [49]. Negative correlation between the powers of delta and beta bands in frontal regions was also shown in highly anxious healthy females performing a social task [50]. In addition, decreases in absolute and relative powers of slow and fast beta were observed in anxious adolescents [51] and in patients with social phobia [52]. Nonetheless, positive delta-beta correlations and decreases in delta power have also been reported in social anxiety disorders but in studies with low-density EEG systems [52, 53]. Spatially, the frontal lobe was the most featured in our PD-anxiety spectral signature. Of interest, disruptions in the prefrontal cortex were consistently reported in neuroimaging studies, characterizing anxiety disorders not only in PD patients [22, 23, 25, 26, 33] but also in non-PD individuals [50, 52, 54].

Regarding the network signature of PD-related anxiety, we have demonstrated that hyper-connectivity networks were mostly dominant in delta, theta, and gamma bands. Previous functional connectivity studies have associated increased severity of anxiety in PD patients with increased functional connectivity between cortical regions of the orbito-frontal cortex and both the inferior-middle temporal and parahippocampal gyri [28] as well as between the insular lobe and both the prefrontal, and cingulate cortices [33]. These findings support the manifestation of the insula, the caudodorsal region of the cingulate gyrus, and the regions within the temporal and frontal lobes as well as their interactions as the most implicated in the hyper-connectivity networks of our results. Indeed, the insula along with the dorsal anterior cingulate (limbic) cortex and the medial prefrontal cortex are all parts of the fear/anxiety circuitry [55] and activations and abnormalities in those regions have been consistently reported in different types of anxiety disorders in the general population [56–59] and in PD subjects [21, 25, 33]. This can be interpreted by the pivotal role of these core regions in processing fear, negative affect, worrisome thoughts and emotions [60–62]. Additionally, hyperconnectivity between subcortical regions, mainly the amygdala and the putamen, and cortical regions of the fear/anxiety circuitry were also persistently associated with anxiety in PD in previous studies [21, 28, 63].

Furthermore, we observed hypo-connectivity networks in alpha and beta bands, predominantly in the frontal and insular lobes. Consistent with our findings, previous research has shown that patterns of decreased connectivity within the frontal lobe are indicative of anxiety in PD patients [25, 28]. Moreover, functional dysconnectivity within and between the salience network, which involves mainly the insular lobe, has also been reported to reflect anxiety disorders in non-PD individuals [57, 64–66].

Importantly, our hypo/hyper-connectivity networks were also shown to be associated with the clinical traits of anxiety in all participants not only at baseline but also longitudinally after 3 years and 5 years. Their progression, reflected by the progression of our NSS, was consistent with the clinical progression of PD-A and PD-NA patients throughout the disease. This longitudinal association can strongly confirm that our EEG-connectivity markers represent the neural correlates of PD-related anxiety throughout the disease progression and thus highlight their predictive capacity. However, despite this internal-longitudinal validation of our anxiety signature, external validation on an independent cohort is necessary for further endorsement.

Finally, some patients in both PD-A and PD-NA groups were under antidepressant and anxiolytic medications during EEG and neuropsychological assessments sessions. Here, we controlled for this issue by demonstrating that the anxiety and depression medication statuses did not differ significantly between PD groups. Besides, topographic EEG changes reported in generalized anxiety disorders during anxiety treatments [67, 68] suggested decreased spectral power of delta and alpha bands along with increased power of beta band [69–71]. Antidepressant medication [72] has also been shown to reduce slow-wave EEG activity and increase the power in alpha band [73, 74]. Notably, these spectral patterns were not reported in our study to characterize the PD-A group, which included patients taking anxiolytics and antidepressants. Excluding these patients would have been an alternative solution in this study, however this would have reduced the sample size in the PD-A group by half and subsequently restricted our statistical analysis. Nonetheless, our longitudinal analysis has demonstrated that the progression of EEG patterns aligns with the progression of anxiety clinical scores in both PD-A and PD-NA patients. This suggests that our EEG-based anxiety signatures reflect the neural correlates of anxiety in PD throughout the disease independently of the anxiety and depression medication statuses.

To summarize, this is the first case-control longitudinal study, to the best of our knowledge, that utilized resting-state HD-EEG to investigate the neural correlates of anxiety in PD. Our findings suggest that increased fronto-parietal delta power, decreased frontal beta power, as well as band-specific hyper/hypo-connectivity networks, are all EEG-based signatures of PD-related anxiety. We showed that the EEG-connectivity signatures are longitudinally associated and can predict the clinical outcomes of anxiety over the course of the disease. Identifying such non-invasive markers may provide new perceptions into the development of advanced biomarkers. Further research could also establish resting-state HD-EEG as a tool for more accurate prognosis, enabling personalized psychiatric follow-up for PD patients at risk of worsening anxiety over time.

### Supplementary information

Supplementary materials

## Data Availability

The data that supports the findings of this study are available upon reasonable request from the corresponding author. The data are not publicly available due to privacy or ethical restrictions.

## Code availability

The codes that support the findings of this study are available upon reasonable request from the corresponding author. The data are not publicly available due to privacy or ethical restrictions.
