# Correction: Electrophysiological signatures of anxiety in Parkinson’s disease

**DOI:** 10.1038/s41398-024-02908-w

**Published:** 2024-04-16

**Authors:** Sahar Yassine, Sourour Almarouk, Ute Gschwandtner, Manon Auffret, Peter Fuhr, Marc Verin, Mahmoud Hassan

**Affiliations:** 1 MRC Brain Dynamic Unit, Nuffield Department of Clinical Neurosciences, University of Oxford, Oxford, UK; 2 University of Rennes, LTSI - U1099, F-35000 Rennes, France; 3 Behavior & Basal Ganglia, CIC1414, CIC-IT, CHU Rennes, Rennes, France; 4 Neuroscience Research Centre, Lebanese University, Faculty of Medicine, Beirut, Lebanon; 5 Department of Neurology, Hospitals of the University of Basel, Basel, Switzerland; 6 Institut des Neurosciences Cliniques de Rennes (INCR), Rennes, France; 7 France Développement Electronique, Monswiller, France; 8 Movement Disorders Unit, Neurology Department, Pontchaillou University Hospital, Rennes, France; 9 School of Science and Engineering, Reykjavik University, Reykjavik, Iceland; 10 MINDIG, F-35000 Rennes, France

**Keywords:** Neuroscience, Psychiatric disorders

Correction to: *Translational Psychiatry* 10.1038/s41398-024-02745-x, published online 27 January 2024

The Funding information section was missing from this article and should have read SNF funded this project (Grant ID: CR3212_159682/1).

