# Supplementary material for: Electrophysiological signatures of anxiety in Parkinson’s disease
Source: Transl Psychiatry. 2024 Jan 27;14:66. doi: 10.1038/s41398-024-02745-x (PMC10821912; doi:10.1038/s41398-024-02745-x)
Supplement: Supplementary file 1 — Supplementary materials [file 41398_2024_2745_MOESM1_ESM.docx]

**Supplementary Materials**

**Electrophysiological signatures of anxiety in Parkinson’s disease**

Sahar Yassine^1,2,3,*^ PhD, Sourour Almarouk^2,3,4,*^ MSc, Ute Gschwandtner^5^ MD, Manon Auffret^2,3,6,7^ PharmD, PhD, Peter Fuhr^5,†^ MD, Marc Verin^2,3,6,8,†^ MD, Mahmoud Hassan^3,9,10,†^ PhD

1 MRC Brain Dynamic Unit, Nuffield Department of Clinical Neurosciences, University of Oxford, Oxford, United Kingdom

2 University of Rennes , LTSI - U1099, F-35000 Rennes, France

3 Behavior & Basal Ganglia, CIC1414, CIC-IT, CHU Rennes, Rennes, France

4 Neuroscience Research Centre, Lebanese University, Faculty of Medicine, Beirut, Lebanon

5 Dept. of Neurology, Hospitals of the University of Basel, Basel, Switzerland

6 Institut des Neurosciences Cliniques de Rennes (INCR), Rennes, France

7 France Développement Electronique, Monswiller, France

8 Movement Disorders Unit, Neurology Department, Pontchaillou University Hospital, Rennes, France

9 School of Science and Engineering, Reykjavik University, Reykjavik, Iceland

10 MINDIG, F-35000, Rennes, France

**^*,†^ These authors contributed equally to this work.**

**Corresponding author:** Sahar Yassine, Ph.D., email: [saharyassine94@gmail.com](mailto:saharyassine94@gmail.com)

**Table S1- Demographic, clinical and main neuropsychiatric characteristics of the study cohort longitudinally expressed as: mean (standard deviation).** y: years, M/F: Male/Female, MoCA: Montreal Cognitive Assessment, MCI (Y/N): Mild Cognitive Impairment (yes/no), UPDRS-III: Unified Parkinson’s Disease Rating Scale-motor examination, LEDD: Levodopa Equivalent Daily Dose, BAI: Beck Anxiety Inventory score, BDI-II: Beck Depression Inventory, second edition score, AES: Apathy Evaluation Scale.

|  | **Baseline** | | **3 years** | | **5 years** | |
| --- | --- | --- | --- | --- | --- | --- |
|  | **PD**  **(N=68)** | **HC**  **(N=25)** | **PD**  **(N=42)** | **HC**  **(N=17)** | **PD**  **(N=34)** | **HC**  **(N=3)** |
| ***Demographic*** |  |  |  |  |  |  |
| Age (y) | 66.4 (8.3) | 66.6 (4) | 70.5 (7.9) | 68.9 (6.1) | 71.1 (6.8) | 65.7 (4.1) |
| Sex (M/F) | 46/22 | 15/10 | 28/14 | 8/9 | 21/13 | 2/1 |
| Education (y) | 14.8 (3.1) | 14.2 (2.9) | 14.8 (3.1) | 13.4 (3.2) | 14.4 (3.1) | 11 (2) |
| ***Clinical*** |  |  |  |  |  |  |
| Disease duration (y) | 5.2 (5.2) | - | 7.5 (4.7) | - | 10.8 (5.1) | - |
| MoCA (/30) | 26 (2.4) | 26.6 (2.7) | 25.2 (3.6) | 27.2 (2.3) | 25.7 (3.9) | 23.3 (4.6) |
| MCI (Y/N) | 22/46 | - | 15/27 | - | 10/24 | - |
| UPDRS-III | 14.8 (11.2) | - | 20.1 (12) | - | 20.3 (13) | - |
| ***Medication*** |  |  |  |  |  |  |
| LEDD (mg/day) | 652 (465) | - | 667 (436) | - | 583 (331) | - |
| ***Neuropsychiatric tests*** |  |  |  |  |  |  |
| BAI (/63) | 9.9 (7.9) | 2.4 (3.2) | 11.5 (7.4) | 2.2 (2.6) | 9.6 (6.8) | 3.3 (4.9) |
| BDI-II (/63) | 7.7 (4.9) | 2.6 (2.5) | 7.8 (4.8) | 1.9 (1.7) | 6.6 (6.2) | 4 (3.6) |
| AES (/63) | 33(8.6) | 24.1 (5.1) | 31.4 (7.1) | 25.1 (5.7) | 30.1 (7.5) | 27.7 (9) |

**Table S2- Affiliation of the EEG channels to the four lobes of interest**

| **Lobes** | **EEG channels** |
| --- | --- |
| **Frontal lobe** | E2, E3, E4, E5, E6, E7, E8, E11, E12, E13, E14, E15, E16, E17, E19, E20, E21, E22, E23, E24, E26, E27, E28, E29, E30, E33, E34, E35, E36, E38, E39, E40, E41, E42, E43, E47, E48, E49, E50, E51, E55, E56, E57, E58, E61, E62, E63, E64, E68, E69, E194, E195, E196, E197, E198, E202, E203, E204, E205, E206, E207, E210, E211, E212, E213, E214, E215, E220, E221, E222, E223, E224. |
| **Parietal lobe** | E9, E44, E45, E52, E53, E59, E60, E65, E66, E70, E71, E72, E74, E75, E76, E77, E78, E79, E80, E81, E84, E85, E86, E87, E88, E89, E90, E96, E97, E98, E99, E100, E101, E109, E110, E119, E128, E129, E130, E131, E132, E140, E141, E142, E143, E144, E152, E153, E154, E155, E161, E162, E163, E164, E170, E171, E172, E173, E179, E180, E181, E182, E183, E184, E185, E186, E192, E193. |
| **Temporal lobe** | E67, E73, E82, E83, E91, E92, E93, E94, E95, E102, E103, E104, E105, E111, E112, E177, E178, E188, E189, E190, E191, E199, E200, E201, E208, E209, E216, E217, E218, E219, E225, E227, E228, E229, E231, E232, E233, E235, E236, E237, E239, E240, E242, E243, E245, E246, E247, E249, E250, E251, E253, E254, E255, E256. |
| **Occipital lobe** | E106, E107, E108, E113, E114, E115, E116, E117, E118, E120, E121, E122, E123, E124, E125, E126, E127, E133, E134, E135, E136, E137, E138, E139, E145, E146, E147, E148, E149, E150, E151, E156, E157, E158, E159, E160, E165, E166, E167, E168, E169, E174, E175, E176, E187. |

**Table S3- Affiliation of brain regions to the seven lobes of interest**

| **Lobes of Interest** | **Brain regions** |
| --- | --- |
| **Prefrontal cortex (PFC)** | Middle frontal gyrus (L/R) |
|  | Orbito-frontal cortex (L/R) |
|  | Superior frontal gyrus (L/R) |
|  | Inferior frontal gyrus (L/R) |
| **Motorstrip (Mot)** | Precentral gyrus (L/R) |
|  | Paracentral lobule (L/R) |
|  | Postcentral gyrus (L/R) |
| **Parietal lobe (Par)** | Inferior parietal lobule (L/R) |
|  | Superior parietal lobule (L/R) |
|  | Precuneus (L/R) |
| **Temporal network (Tmp)** | Inferior temporal gyrus (L/R) |
|  | Middle temporal gyrus (L/R) |
|  | Superior temporal gyrus (L/R) |
|  | Parahippocampal gyrus (L/R) |
|  | Fusiform gyrus (L/R) |
|  | Posterior superior temporal sulcus (L/R) |
| **Occipital lobe (Occ)** | Lateral occipital cortex (L/R) |
|  | MedioVentral occipital cortex (L/R) |
| **Limbic lobe (Lmb)** | Caudal cingulate gyrus (L/R) |
|  | Ventral cingulate gyrus (L/R) |
|  | Dorsal cingulate gyrus (L/R) |
| **Insular lobe (Ins)** | Insular gyrus (L/R) |


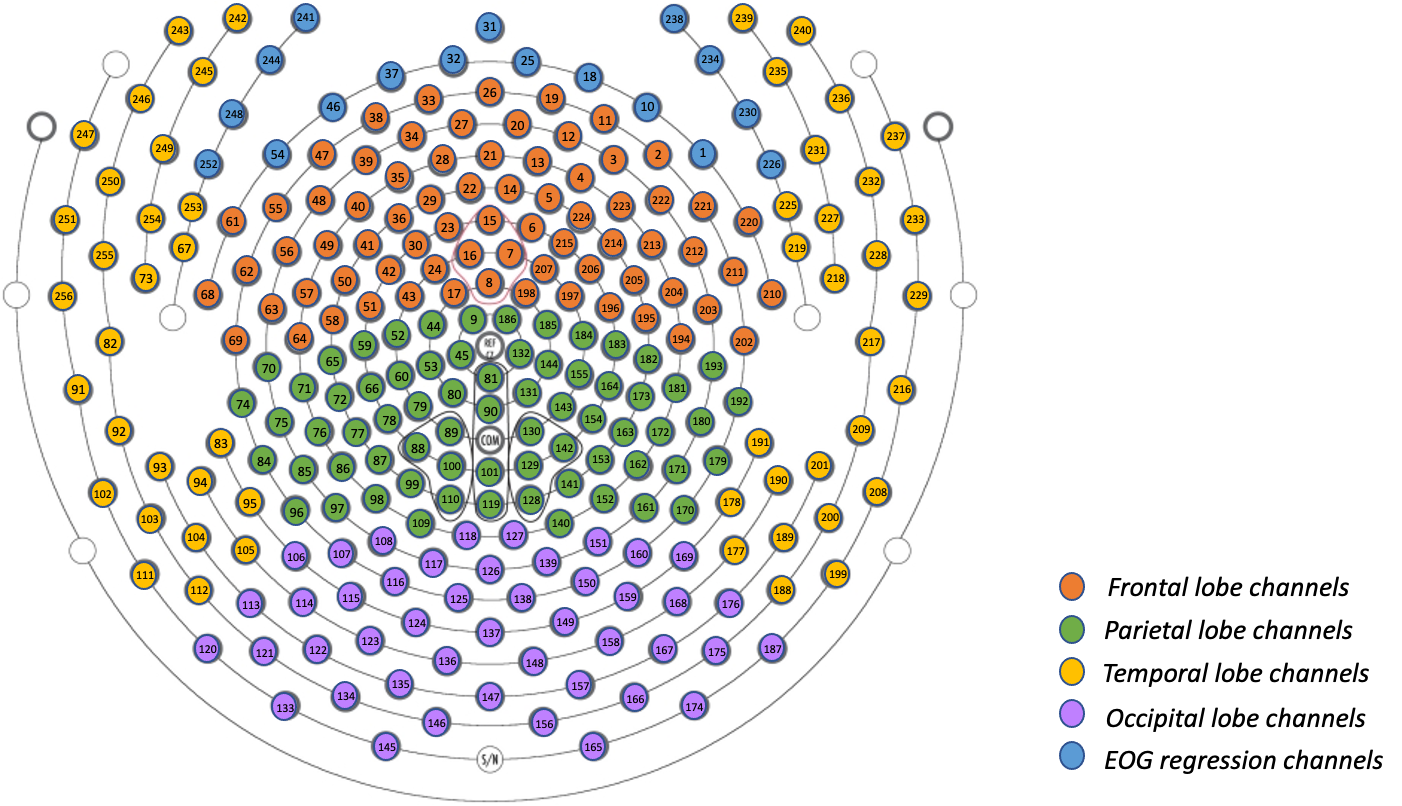


**Figure S1- EGI 256-channels sensor layout and the affiliation of the channels to the four lobes of interest:** Frontal lobe (orange), Parietal lobe (green), temporal lobe (yellow) and occipital lobe (purple). The 17 channels used for EOG regression are marked in blue.


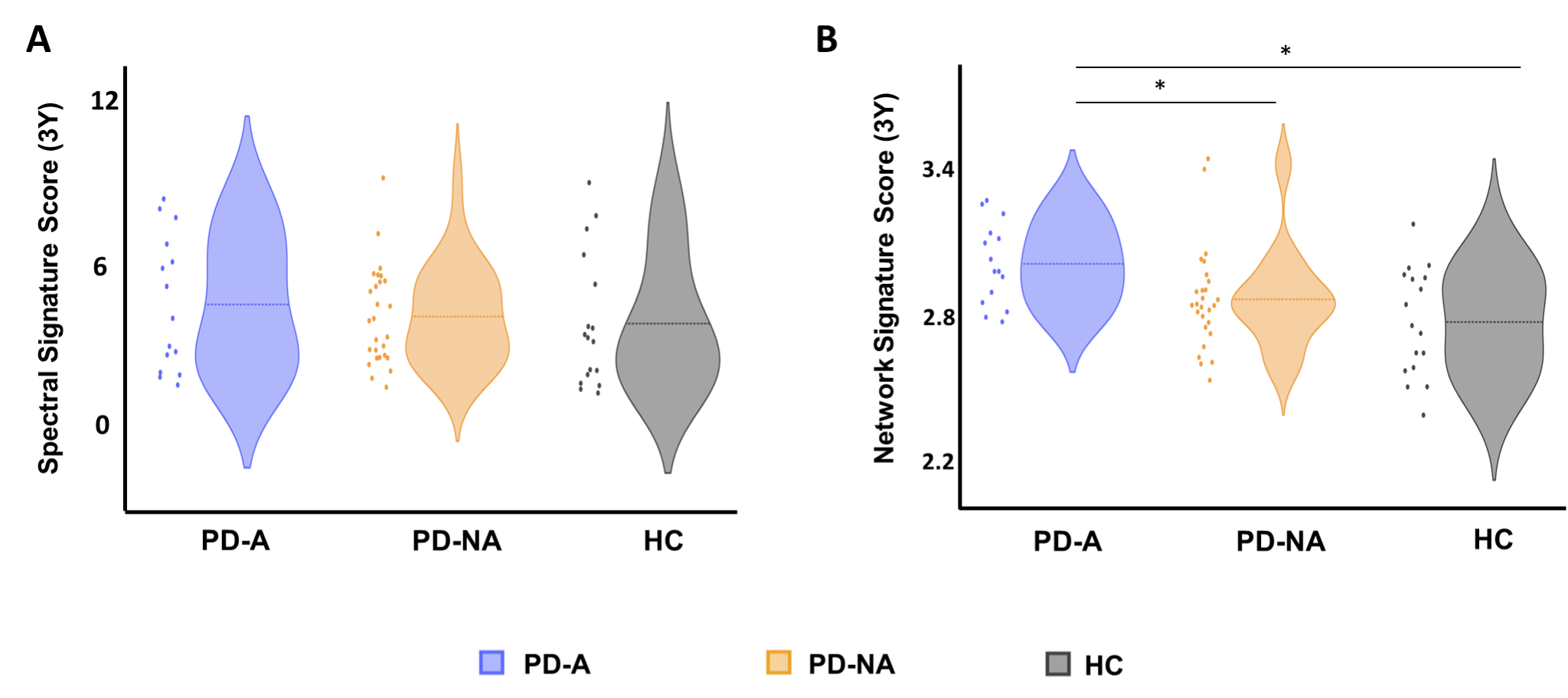


**Figure S2- A) Spectral Signature Score (SSS) and B) Network signature score (NSS) of the three groups computed at 3Y.** * p<0.05 (p-values are corrected using Bonferroni for multiple comparisons).
